# RTeQTL: Real-Time Online Engine for Expression Quantitative Trait Loci Analyses

**DOI:** 10.1093/database/bau066

**Published:** 2014-07-18

**Authors:** Baoshan Ma, Jinyan Huang, Liming Liang

**Affiliations:** ^1^College of Information Science and Technology, Dalian Maritime University, Dalian, Liaoning Province, China 116026, ^2^Department of Epidemiology, Harvard School of Public Health, Boston, MA, USA 02115 and ^3^Department of Biostatistics, Harvard School of Public Health, Boston, MA, USA 02115

## Abstract

Our database tool, called Real-Time Engine for Expression Quantitative Trait Loci Analyses (RTeQTL), can efficiently provide eQTL association results that are not available in existing eQTL databases browsers. These functions include (i) single SNP (single-nucleotide polymorphism) and (ii) two-SNP conditional eQTL effects on gene expression regardless of the magnitude of *P*-values. The database is based on lymphoblastoid cell lines from >900 samples with global gene expression and genome-wide genotyped and imputed SNP data. The detailed result for any pairs of gene and SNPs can be efficiently computed and browsed online, as well as downloaded in batch mode. This is the only tool that can assess the independent effect of a disease- or trait-associated SNP on gene expression conditioning on other SNPs of interest, such as the top eQTL of the same gene. It is also useful to identify eQTLs for candidate genes, which are often missed in existing eQTL browsers, which only store results with genome-wide significant *P*-value. Additional analyses stratifying by gender can also be easily achieved by this tool.

**Database URL:** http://eqtl.rc.fas.harvard.edu/

## Introduction

The ability to interrogate and study the genetics of functional phenotypes that are intermediate between a DNA variant and a disease phenotype of interest can point to the true biological mechanism, critical to disease etiology. Gene expression is one of these key intermediate functional phenotypes (1–3). Numerous studies illuminate significant genetic variation, within and between human populations that affects gene expression levels, and by doing so may underlie phenotypic variation (e.g., 4, 5–8).

Existing databases (such as eqtl.uchicago.edu/cgi- bin/ gbrowse/eqtl/, www.sanger.ac.uk/resources/software/ genevar/, www. scandb.org/newinterface/about.html, www. ncbi.nlm.nih. gov/projects/gap/eqtl/index.cgi, www.hsph.harvard.edu/li ming-liang/software/eqtl/, www.sph.umich.edu/csg/liang/im p u tation/, www.sph.umich.edu/csg/liang/asthma/) of expression quantitative trait loci (eQTLs) based on lymphoblastoid cell lines (LCL) and other tissues (4, 6–20) have helped interpret findings from genome-wide association studies (GWAS) for complex diseases and traits, including childhood asthma, Crohn’s disease, Type 2 diabetes, circulating resistin levels, Graves’ disease, human height, body mass index, waist-hip ratio, osteoporosis-related traits, skin cancer, esophageal squamous-cell carcinoma and human red blood cell. To the best of our knowledge, all public available eQTL browsing tools based on LCL or other tissues only provide significant eQTLs based on stringent GWAS threshold, and all results were based on single variant analysis. However, after identification of disease or trait-associated eQTL using existing eQTL browsers, it is often required to assess whether a disease-associated variant has an independent effect on gene expression after conditioning on the peak eQTL for the same gene (21–24). For candidate gene study, it is also desirable to report eQTLs that pass a less stringent significance cutoff because of the far lower number of multiple tests compared with GWAS of gene expression traits, where billions of hypotheses were tested for cis (local) and trans (distant) eQTLs. All of these analyses are not feasible for existing eQTL browsers without accessing the raw genotype data, which are usually not publicly available. And it is computationally impossible to precompute such analyses for all single-nucleotide polymorphisms (SNPs) and genes on the genome and store the results in any eQTL browser. As human diseases and traits depict sex-specific genetic architecture (25), a sex-specific eQTL assessment would provide unique insight into the etiology of the disease or trait of interest (26). This information is not possible to achieve from existing eQTL databases.

Here, we developed an online database tool that can do all above eQTL analysis in real time without the need to share raw genetic and gene expression data. Specifically, this tool can test for association between any gene expression and any two SNPs chosen by the user. The analyses can be done using the full samples or stratified by male and/or female. Single variant analyses for any pair of gene-SNP are also provided. All results are output to web table format and can be downloaded in batch mode.

### Description

Real-Time Engine for Expression Quantitative Trait Loci Analyses (RTeQTL) is a web-based database tool, and hence all computation is carried out at the server side. A user-friendly interface is provided to facilitate easy access and interpretation of results

#### Data

Gene expression in LCL was characterized in two independent data sets, one sample of 405 siblings using Affymetrix HG U133 Plus 2.0 chips (>54 000 transcription probesets, Medical Research Council asthma study family panel (MRCA) data set (4) and the other sample of 550 siblings using Illumina Human6 V1 array (>47 000 transcription probes, Medical Research Council eczema study family panel (MRCE) data set). All samples include Caucasians of British descendant. Among these individuals, 928 were also genotyped at >300 000 SNPs using the Illumina HumanHap300 arrays, with additional genotypes for 2 million SNPs in the HapMap Project filled in using imputation. These two data sets together identified genome-wide significant *cis* and *trans* eQTLs for 14 177 genes (27). We will impute the latest version of 1000 Genome Project variants whenever available and update the Web site.

#### Microarray hybridization and normalization

The peripheral blood lymphocytes were transformed by Epstein-Barr virus, and then cultured in 500-ml roller. The cell lines were collected when the cell lines reached the log phase followed by storing at –80°C until use. RNA was extracted from the samples stored at −80°C in batches using the RNeasy Maxi Kit, after which the quality and the quantity of RNA were evaluated. In all, 10 mg of RNA was used to synthesize cDNA, which was used as a template *in vitro* transcription according to the manufacturer’s instruction. Then 15 mg of labeled, fragmented cRNA was hybridized to Affymetrix U133 Plus 2.0 GeneChips and Illumina Human6 V1 array for MRCA and MRCE data sets, respectively (27).The MRCA expression data were normalized using the robust multi-array average package to remove any technical or spurious background variation. The MRCE expression data were normalized using quantile normalization based on expression values from GenomeStudio.

#### Whole-genome genotyping and imputation

All DNA samples were subjected to stringent quality control to check for fragmentation and amplification. We adopted 20 ml of DNA at a concentration of 50 ng/ml for each array. Whole-genome genotyping was performed according to manufacturers’ protocol using the Illumina HumanHap300 Genotyping BeadChip in a BeadLab with full automation, and the process was traced in real time. We excluded SNPs with call rate <95%, Hardy–Weinberg equilibrium *P* < 10^−6^ and MAF <2%. We imputed genotypes from all HapMap2 SNPs using Markov chain haplotyping (MaCH) package (28). All imputed SNPs with low imputation quality score (Rsquare<0.3) were excluded from the database.

#### Statistical analysis model

Linear mixed model is used to account for the family relatedness in the data set. For the sibling data, this model is identical to the model implemented in the multipoint engine for rapid likelihood inference (MERLIN) package (29) that was used in previous publication on the same data sets (4, 27). Specifically, the expression level of an expression probe is modeled as:

(1) $$\text{probe}=\alpha +\text{SNP}1\cdot \beta_{1}+\text{SNP}2\cdot \beta_{2}+\text{Z}+\text{ε}$$

where $\beta_{1}$ is the fixed effect for SNP1 and $\beta_{2}$ is the fixed effect for SNP2, *Z* is random effect for family and ε is residual error. R package nlme is used to fit this model and test the SNP effect. The same model excluding the term for SNP2 is used to do single SNP analysis. For analyses stratified by sex, this model is applied to male or female separately. Before fit model (1), inverse normal transformation was applied to expression level to remove outlier’s effect, and batch effects were removed by adjusting principal components calculated based on all genes expression (13, 27).

#### User input

**Figure 1.**
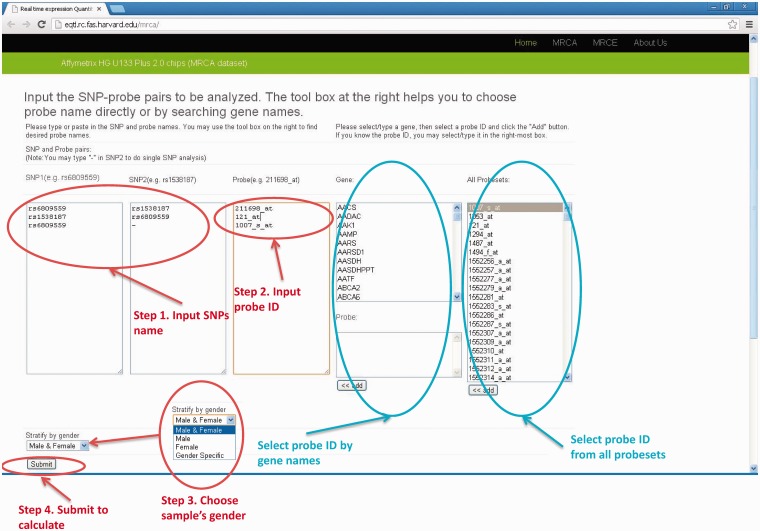
The input web page (MRCA example). There are four steps to submit a query. (1) input SNP1 and SNP2 names (2) input probe ID (3) select sample's gender (4) click the “Submit” button.

The user chooses the gene and SNPs in analysis. The probe name for either the Affymetrix or Illumina platform can be chosen by specifying gene names and then adding them to the input box for probes. For analyses involving multiple pairs of gene and SNPs (batch mode), the list of SNP rs names and probe names can be copied and pasted into the corresponding input boxes. There are two cases for SNP columns: (i) when single SNP analysis is desirable, the user inputs the SNP rs name into ‘SNP1’ column and ‘-’ (short dash) in the ‘SNP2’ column. (ii) When two SNPs analyses are desirable, the user inputs the SNP1 rs name into ‘SNP1’ column and SNP2 rs name in the ‘SNP2’ column. When analyses stratified by sex are needed, the user can choose appropriate data sets in the drop-down menu named ‘Stratify by gender’, where ‘Male & Female’ means analysis using full samples without considering SNP*gender interaction effect, ‘Male” or “Female” will only output results for male or female data set, respectively, and ‘Gender Specific’ will perform analysis in male and female separately but output both results. Sanity check for input names will also be performed. We provide a manual on our website and readers will find detailed description on how to use our database http://eqtl.rc.fas.harvard.edu/mrce/static/RTeQTL_manual_20130623.pdf. For example, the input setting shown in Figure 1 will return eQTL results for the following three models:

Expression of 211698_at = rs6809559 + rs1538187 (Two-SNP analysis)

Expression of 121_at = rs1538187 + rs6809559 (Two-SNP analysis)

Expression of 1007_s_at = rs6809559 (Single-SNP analysis)

#### Results output

**Figure 2.**
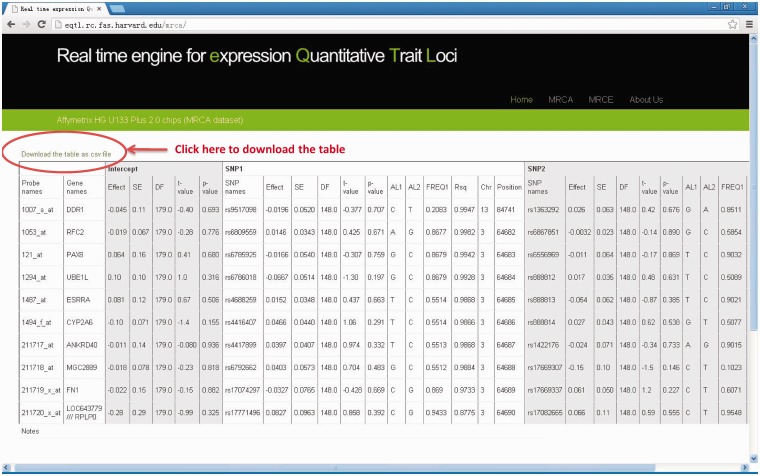
The output web page (MRCA example). Full details for regression results are available on output webpage and results table can be downloaded to desktop computer by clicking the link “Download the table as csv file”.

Results table will be output on the web page and can be downloaded to desktop computer by clicking the link ‘Download the table as csv file’ on top of the result table. Each row corresponds to a result for each pair of gene and SNPs. Full details for regression results are available, including effect size, standard error, test statistics, *P*-value as well as gene annotation of the expression probe and SNPs (chromosomal position, allele label, allele frequency, MaCH imputation quality score, Rsq- see Table 1) and the column to indicate the samples used for analysis. If we compute single SNP, the corresponding outputs of the SNP2 are ‘NA’. If the input SNP or probe name could not be found in our data files, there will be some notes in the last row of the output table. See Figure 2 for an output example.

**Table 1.** Headers and description of the online output table

Column order	Name	Description
1	Effect	Effect size $\hat{\beta }$ from linear mixed model. The amount of increase/decrease expression by one copy of the Allele 1 in the unit of one standard deviation
2	SE	Standard error of $\hat{\beta }$
3	DF	Degrees of freedom of the test
4	*t*-value	$\hat{\beta }$/SD($\hat{\beta }$)
5	*P*-value	The probability of P{t > |T|}
6	AL1/2	The allele1/2 label
7	FREQ1	Frequency for Allele1
8	Chr	Chromosome
9	Position	Position on chromosome (NCBI 36)
10	Rsq	MaCH imputation quality score, which estimates the squared correlation between imputed and true allele counts
11	Gender	Sample’s gender when analysis stratified by gender

This table provides the header names and description of the columns of the result table for online association analyses output by the RTeQTL website.

### Implementation

Python and HTML languages are used to control workflow and provide efficient access to the data. R function is used to compute the linear mixed model. Original SNP data and expression data were deidentified and stored as binary format. We built efficient index so that specific SNP and expression data can be retrieved in real time. Specifically, one design feature of this engine is that we transform the huge text files of SNPs and gene expression data into multiple smaller binary files to accelerate I/O reading speed. The other feature of this engine is that we used hierarchical index so that the SNPs data corresponding to the SNPs name input from web page can be quickly located and acquired in the binary files. The analysis for 100 gene-SNPs pairs takes only 20s.

### Examples

Our eQTL database has been applied to real biological data. The first example is for analysis stratified by gender (26). A genome-wide search for sexually dimorphic associations with height, weight, body mass index, waist circumference, hip circumference and waist-hip ratio was conducted and results demonstrate the value of sex-stratified GWAS to unravel sexually dimorphic genetic underpinning complex traits. The other example is for eQTL conditional analysis (21–24). The conditional analyses were performed for all expression data, except for cortical tissue, by conditioning the trait-associated SNP on the most significant *cis*-associated SNP for that particular gene transcript and vice versa.

### Commitment to future updates

We will impute genetic variants from the 1000 Genomes panel (phase 1) and update the database. Each following release of 1000G variants will be imputed and incorporated to the database.

## Conclusion and Discussion

We developed an efficient web-based database tool for eQTL analysis of any gene and SNPs available. Both single-SNP and two-SNP analyses can be performed, as well as analyses stratified by males and females. The computational result for any pairs of gene and SNPs can be shown online and downloaded in comma separate values (CSV) format. Controlling for multiple testing is important even for candidate gene study. The number of tests to control is determined by the actual number of SNP-gene pairs queried from the Web site, instead of the number of available SNPs around the locus of interest. As a general guideline to provide a sense of significance level at genome-wide average, we note that we previously estimated that 5% false discovery rate (FDR) accounting for all cis and trans pairs corresponded to *P* < 1.02 × 10^−7^ (1% FDR corresponding to *P* < 1.62 × 10^−8^) (27). For *cis* eQTL defined as SNP and probe within 1 Mb of each other, the 1% FDR corresponded to *P* < 6.83 × 10^−5^.

To the best of our knowledge, it is the only online tool that can evaluate the independent effect of a disease- or trait-associated SNP on gene expression conditioning on other SNPs of interest, such as the top eQTL of the same gene. We commit to update the web tool regularly by incorporating more gene expression data sets and imputing the latest panel of variants from the 1000 Genomes Project when available.

## References

[1] Cheung V.G. Spielman R.S. (2009) Genetics of human gene expression: mapping DNA variants that influence gene expression. Nat. Rev. Genet., 10, 595–604 19636342 10.1038/nrg2630 PMC2989458

[2] Cookson W. Liang L. Abecasis G.*.* (2009) Mapping complex disease traits with global gene expression. Nat. Rev. Genet., 10, 184–194 19223927 10.1038/nrg2537 PMC4550035

[3] Nica A.C. Dermitzakis E.T. (2008) Using gene expression to investigate the genetic basis of complex disorders. Hum. Mol. Genet., 17, R129–R134 18852201 10.1093/hmg/ddn285 PMC2570059

[4] Dixon A.L. Liang L. Moffatt M.F.*.* (2007) A genome-wide association study of global gene expression. Nat. Genet., 39, 1202–1207 17873877 10.1038/ng2109

[5] Goring H.H. Curran J.E. Johnson M.P.*.* (2007) Discovery of expression QTLs using large-scale transcriptional profiling in human lymphocytes. Nat. Genet., 39, 1208–1216 17873875 10.1038/ng2119

[6] Zeller T. Wild P. Szymczak S.*.* (2010) Genetics and beyond—the transcriptome of human monocytes and disease susceptibility. PLoS One, 5, e10693 20502693 10.1371/journal.pone.0010693 PMC2872668

[7] Stranger B.E. Nica A.C. Forrest M.S.*.* (2007) Population genomics of human gene expression. Nat. Genet., 39, 1217–1224 17873874 10.1038/ng2142 PMC2683249

[8] Dimas A.S. Deutsch S. Stranger B.E.*.* (2009) Common regulatory variation impacts gene expression in a cell type-dependent manner. Science, 325, 1246–1250 19644074 10.1126/science.1174148 PMC2867218

[9] Veyrieras J.B. Kudaravalli S. Kim S.Y.*.* (2008) High-resolution mapping of expression-QTLs yields insight into human gene regulation. PLoS Genet., 4, e1000214 18846210 10.1371/journal.pgen.1000214 PMC2556086

[10] Degner J.F. Pai A.A. Pique-Regi R.*.* (2012) DNase I sensitivity QTLs are a major determinant of human expression variation. Nature, 482, 390–394 22307276 10.1038/nature10808 PMC3501342

[11] Schadt E.E. Molony C. Chudin E.*.* (2008) Mapping the genetic architecture of gene expression in human liver. PLoS Biol, 6, e107 18462017 10.1371/journal.pbio.0060107 PMC2365981

[12] Myers A.J. Gibbs J.R. Webster J.A.*.* (2007) A survey of genetic human cortical gene expression. Nat Genet, 39, 1494–1499 17982457 10.1038/ng.2007.16

[13] Pickrell J.K. Marioni J.C. Pai A.A.*.* (2010) Understanding mechanisms underlying human gene expression variation with RNA sequencing. Nature, 464, 768–772 20220758 10.1038/nature08872 PMC3089435

[14] Gaffney D. Veyrieras J.B. Degner J.*.* (2012) Dissecting the regulatory architecture of gene expression QTLs. Genome Biol., 13, R7 22293038 10.1186/gb-2012-13-1-r7 PMC3334587

[15] Innocenti F. Cooper G.M. Stanaway I.B.*.* (2011) Identification, replication, and functional fine-mapping of expression quantitative trait loci in primary human liver tissue. PLoS Genet., 7, e1002078 21637794 10.1371/journal.pgen.1002078 PMC3102751

[16] Montgomery S.B. Sammeth M. Gutierrez-Arcelus M.*.* (2010) Transcriptome genetics using second generation sequencing in a Caucasian population. Nature, 464, 773–777 20220756 10.1038/nature08903 PMC3836232

[17] Grundberg E. Small K.S. Hedman A.K.*.* (2012) Mapping cis- and trans-regulatory effects across multiple tissues in twins. Nat. Genet., 44, 1084–1089 22941192 10.1038/ng.2394 PMC3784328

[18] Stranger B.E. Montgomery S.B. Dimas A.S.*.* (2012) Patterns of cis regulatory variation in diverse human populations. PLoS Genet., 8, e1002639 22532805 10.1371/journal.pgen.1002639 PMC3330104

[19] Nica A.C. Parts L. Glass D.*.* (2011) The architecture of gene regulatory variation across multiple human tissues: the MuTHER study. PLoS Genet., 7, e1002003 21304890 10.1371/journal.pgen.1002003 PMC3033383

[20] Grundberg E. Meduri E. Sandling J.K.*.* (2013) Global analysis of DNA methylation variation in adipose tissue from twins reveals links to disease-associated variants in distal regulatory elements. Am. J. Hum. Genet., 93, 876–890 24183450 10.1016/j.ajhg.2013.10.004 PMC3824131

[21] Lango Allen H. Estrada K. Lettre G. (2010) Hundreds of variants clustered in genomic loci and biological pathways affect human height. Nature, 467, 832–838 20881960 10.1038/nature09410 PMC2955183

[22] Berndt S.I. Gustafsson S. Magi R. (2013) Genome-wide meta-analysis identifies 11 novel loci for anthropometric traits and provides new insights on the genetic architecture of the extremes of the distribution. *Nat. Genet*., 45, 501–512 23563607 10.1038/ng.2606 PMC3973018

[23] Speliotes E.K. Willer C.J. Berndt S.I.*.* (2010) Association analyses of 249, 796 individuals reveal 18 new loci associated with body mass index. Nat. Genet., 42, 937–948 20935630 10.1038/ng.686 PMC3014648

[24] Heid I.M. Jackson A.U. Randall J.C.*.* (2010) Meta-analysis identifies 13 new loci associated with waist-hip ratio and reveals sexual dimorphism in the genetic basis of fat distribution. Nat Genet, 42, 949–960 20935629 10.1038/ng.685 PMC3000924

[25] Ober C. Loisel D.A. Gilad Y. (2008) Sex-specific genetic architecture of human disease. Nat. Rev. Genet., 9, 911–922 19002143 10.1038/nrg2415 PMC2694620

[26] Randall J.C. Winkler T.W. Kutalik Z.*.* (2013) Sex-stratified genome-wide association studies including 270,000 individuals show sexual dimorphism in genetic loci for anthropometric traits. *PLoS Genet*., 9, e1003500 23754948 10.1371/journal.pgen.1003500 PMC3674993

[27] Liang L. Morar N. Dixon A.L.*.* (2013) A cross-platform analysis of 14 177 expression quantitative trait loci derived from lymphoblastoid cell lines. Genome Res., 23, 716–726 23345460 10.1101/gr.142521.112 PMC3613588

[28] Li Y. Willer C.J. Ding J.*.* (2010) MaCH: using sequence and genotype data to estimate haplotypes and unobserved genotypes. Genet. Epidemiol., 34, 816–834 21058334 10.1002/gepi.20533 PMC3175618

[29] Chen W.M. Abecasis G.R. (2007) Family-based association tests for genomewide association scans. Am. J. Hum. Genet., 81, 913–926 17924335 10.1086/521580 PMC2265659

